# A Numerical Procedure for Shakedown Analysis of Thick Cylindrical Vessels with Crossholes under Dual Cyclic Loadings

**DOI:** 10.3390/ma16093364

**Published:** 2023-04-25

**Authors:** Yangxi Chen, Xin Jin, Yan Guo, Jian Zhao, Sujuan Guo

**Affiliations:** 1 Key Laboratory of Pressure Systems and Safety, Ministry of Education, East China University of Science and Technology, Shanghai 200237, China; 2 China Nuclear Power Technology Research Institute Co., Ltd., Shenzhen 518000, China; 3 Sino-French Institute of Nuclear Engineering and Technology, Sun Yat-sen University, Guangzhou 510275, China; 4 School of Aerospace Engineering and Applied Mechanics, Tongji University, Shanghai 200092, China; 5 Shanghai Institute of Aircraft Mechanics and Control, Shanghai 200092, China

**Keywords:** shakedown, cyclic internal pressure, cyclic thermal loading, thick cylindrical vessels, crossholes

## Abstract

A modified numerical procedure for the shakedown analysis of structures under dual cyclic loadings, based on the Abdalla method, is proposed in this paper. Based on the proposed numerical procedure, the shakedown analysis of the thick cylindrical vessels with crossholes (TCVCs) under cyclic internal pressure and cyclic thermal loading was carried out. The effects of material parameters (elastic modulus and thermal expansion coefficient) and crosshole radius on the elastic shakedown limit of TCVCs are discussed and, finally, normalized and formularized. Furthermore, the obtained shakedown limit boundary formulation is compared with FEA results and is verified to evaluate the shakedown behavior of TCVCs under cyclic internal pressure and cyclic thermal loading.

## 1. Introduction

**Figure 1:**
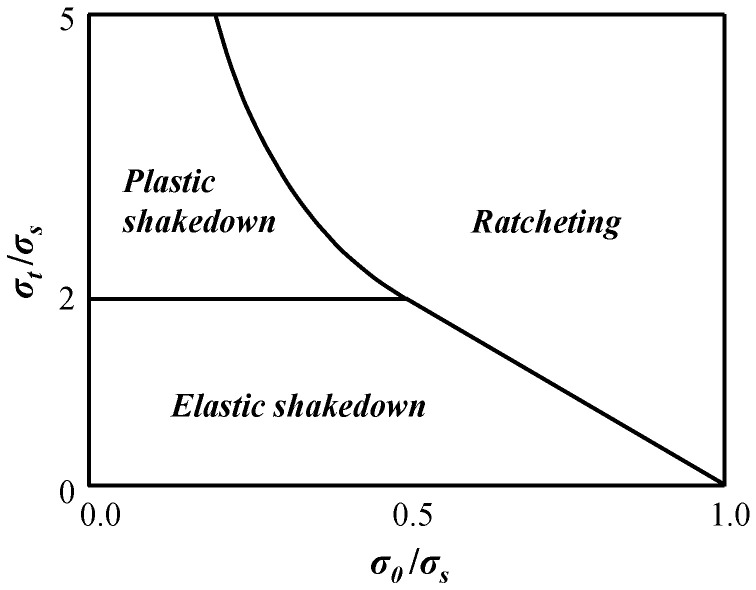
Typical Bree diagram.

Pressure-bearing components in petrochemical and nuclear power plants are usually operated under cyclic loadings. Throughout their lifetime, pressure-bearing components may face the problem of reversed plasticity or ratcheting failure. Therefore, it is very important to establish an effective shakedown analysis method to prevent early failure due to reversed plasticity or ratcheting; the corresponding Bree loading zoning diagram is shown in Figure 1, where *σ*_0_ denotes the mechanical stress, *σ*_t_ is the thermal stress, and *σ*_s_ is the yield stress [1].

With the increasing emphasis on shakedown analysis in engineering, the theoretical study of the classical upper and lower bound shakedown theorems has gradually matured, and the corresponding numerical algorithms for shakedown analysis have also been rapidly developed [1,2,3,4,5,6,7,8,9,10,11,12,13,14]. The researchers have proposed some shakedown evaluation methods, such as the cycle-by-cycle method (CBC) [15,16,17], elastic compensation method (ECM) [2,3,4], and nonlinear superposition methods (Muscat and Mackenzie method, min{*P_L_*, 2*P_e_*} method, and Abdalla method) [5,6,7,8,9,10,11]. The CBC method has higher accuracy in shakedown assessment, but it is very time-consuming, so it is mainly used for verification of other shakedown analysis methods. The ECM method calculates the shakedown limits through the elastic finite element iteration, and each iteration needs to adjust the elastic modulus of the elements to obtain the redistribution of stress. The ECM method can achieve high accuracy for simple structures, but it often has a large calculation error for complex structures [18,19]. The Muscat and Mackenzie method can only be used for shakedown analysis of structures under mechanical loadings, while the min{*P_L_*, 2*P_e_*} method just supports the proportional loadings. It is necessary to find a simple and accurate method applicable to structures subjected to thermomechanical loadings as shown in Table 1. Furthermore, in the existing studies [1,2,3,4,5,6,7,8,9,10,11,12,13,14,15,16,17,18,19,20,21,22,23,24] considerable research has been undertaken on the shakedown of pressure-bearing structures under constant pressure and cyclic thermal loading, and those considering the shakedown behavior of TCVC and other structures under the cyclic pressure and cyclic thermal loading simultaneously (dual cyclic loadings) have not been reported. The establishment of a numerical flow for calculating the shakedown analysis of structures under dual cyclic loadings is crucial.

Thick cylindrical vessels are one kind of important pressure-bearing component in the process equipment. Meanwhile, thick cylindrical vessels often need crossholes to ensure equipment maintenance, material transfer, and connection between devices [25,26]. During service, thick cylindrical vessels with crossholes are often subjected to simultaneous cyclic pressure and cyclic temperature changes due to equipment shutdowns, startups, and peak regulation. In addition, the introduction of crossholes changes the original stress distribution of the structures, which causes significant stress concentration [27]. The structures are then prone to losing their initial shakedown state and may undergo reversed plasticity or ratcheting failure. Therefore, it is vital to carry out research on safety analysis of thick cylindrical vessels with crossholes (TCVCs) under dual cyclic loadings.

In summary, there is an urgent need for the development of a shakedown analysis procedure for the TCVC under dual cyclic loadings in which the influencing factors of material properties and structural dimensions are considered. In this paper, the obtained shakedown limits of four classical methods, namely, the min{*P_L_*, 2*P_e_*} method, the Abdalla method, the stress analysis design method (SAD), and the cycle-by-cycle method (CBC), are first introduced and a precision comparison is carried out. According to the comparison results, a modified numerical procedure for the shakedown analysis of structures under dual cyclic loadings is established based on the Abdalla method by making a distinction between constant loadings and cyclic loadings and introducing the loading ratios. Then, considering the effects of material parameters (elastic modulus and thermal expansion coefficient) and crosshole radius, the shakedown limits of the TCVC under dual cyclic loadings are studied and formularized based on the proposed numerical procedure, which was verified to be effective. The research results have important reference and guiding significance for studying the shakedown behavior of structures under dual cyclic loadings.

## 2. Classical Methods for Shakedown Analysis

### 2.1. Cycle-by-Cycle Method

The cycle-by-cycle method is the most fundamental technique for determining the shakedown limit of structures, and it is frequently employed to examine the effectiveness of other shakedown analysis methods [15]. Using the cycle-by-cycle method, the finite element method is used to simulate the stress–strain behavior of structures under cyclic loadings. The shakedown behavior of structures is judged according to the convergence of accumulated plastic strain, and the approximate solution of the shakedown limit load is obtained by the loading approximation method. Using the cycle-by-cycle method, Zheng et al. investigated the shakedown limit of thick cylinders with radial openings subjected to thermomechanical loadings [16]. Camilleri et al. investigated the shakedown and ratcheting behavior of a thin cylinder, a thick cylinder, and a thick cylinder with a radial crosshole [17].

The cycle-by-cycle method is applicable to structures under non-proportional loadings. It requires a considerable number of cycles and a series of loading combinations to find the shakedown limit load, which greatly affects the efficiency of the calculation. Therefore, this method is often used to check the accuracy of other methods.

### 2.2. min{P_L_, 2P_e_} Method

For the min {*P_L_*, 2*P_e_*} method, the elastic shakedown limit of a structure under proportional loadings can be further simplified to the lesser of the limit load and twice the elastic limit load. The limit load *P_L_* of the structure is determined according to the elastic-perfectly plastic analysis, while the elastic limit *P_e_* of the structure is determined according to the yield strength *σ_s_* of the material and the maximum elastic equivalent stress ${\left|\sigma_{ei}\right|}_{\max}$ of the structure under any proportional loading *P_i_*:

(1) $$2P_{e}=2\sigma_{s}\cdot P_{i}/{\left|\sigma_{ei}\right|}_{\max}$$

The elastic shakedown limit *P*_s_ is:

(2) $$P_{s}=\min\left\{P_{L},2P_{e}\right\}$$

The min {*P_L_*, 2*P_e_*} method is convenient in calculation, but it can only be used to determine the shakedown limit of structures under proportional loadings.

### 2.3. Abdalla Method

Abdalla split the operating loading under non-proportional loading into two parts: constant loading and cyclic loading, and proposed a simplified method to determining the shakedown limit of the structures [9]. The simplified method utilizes small displacement formulation and determines the shakedown limit load by performing elastic analysis and elastic-plastic analysis. In the elastic analysis, only the cyclic loading type is applied individually to obtain the elastic stress field $\sigma_{E}$ of the structures. In the elastic-plastic analysis, both the constant and cyclic loading are applied in two consecutive analysis steps to obtain the elastic-plastic stress field $\sigma_{EPi}$ of the structures. Finally, the two stress fields are superimposed to obtain the maximum residual stress field satisfying the Melan shakedown theorem:

(3) $$\sigma_{ri}=\sigma_{EPi}-\sigma_{E}T_{i}/T_{N}$$

(4) $$\sigma_{r}^{eq}=\frac{1}{\sqrt{2}}{\left[{\left(\sigma_{rx}-\sigma_{ry}\right)}^{2}+{\left(\sigma_{ry}-\sigma_{rz}\right)}^{2}+{\left(\sigma_{rz}-\sigma_{rx}\right)}^{2}+6\left(\tau_{rxy}^{2}+\tau_{ryz}^{2}+\tau_{rzx}^{2}\right)\right]}^{1/2}$$

The shakedown limit load is the loading that corresponds to the residual stress field. Abdalla et al. applied this method to solve two benchmark shakedown problems, namely, the two-bar structure subjected to constant axial force and cyclic thermal loading, and the Bree cylinder subjected to constant internal pressure and cyclic high-temperature variation across its wall [10]. The advantages of the Abdalla method are high accuracy and efficiency, while the method is also applicable to structures under non-proportional loadings.

### 2.4. Stress Analysis Design Method

In the ASME Boiler and Pressure Vessel Code Sec. VIII Division 2, the stress analysis design method is supplied [28]. Using this method, the maximum shear stress theory is adopted, and the stress is divided into primary stress, secondary stress, and peak stress based on the location of stresses, loading conditions, stress properties, and other factors. Also in the stress analysis design method, the stress intensity checks are classified into five categories according to the stress conditions in different paths of the pressure vessel, which are listed as follows:

(5) $$P_{m}\leq [\sigma ]$$

(6) $$P_{L}\leq 1.5[\sigma ]$$

(7) $$P_{L}+P_{b}\leq 1.5[\sigma ]$$

(8) $$P_{L}+P_{b}+Q\leq 3[\sigma ]$$

(9) $$P_{L}+P_{b}+Q+F\leq S_{a}$$

where *P_m_* is the primary membrane stress, *P_L_* means the local primary membrane stress, *P_b_* is the primary bending stress, *Q* is the secondary stress, *F* is the peak stress, and *S_a_* is the fatigue strength. The stress analysis design method can be used to quickly judge whether the common component shakes down or not; however, in complex engineering practice, there are still disputes in how to classify the stresses.

In summary, the Abdalla method has higher accuracy and generality. In this study, the Abdalla method is further developed for dual cyclic loadings in the proposed shakedown analysis procedure of the TCVC, as introduced in the following section.

## 3. The Proposed Numerical Procedure and Finite Element Model

### 3.1. The Modified Shakedown Analysis Procedure for Dual Cyclic Loadings

**Figure 2:**
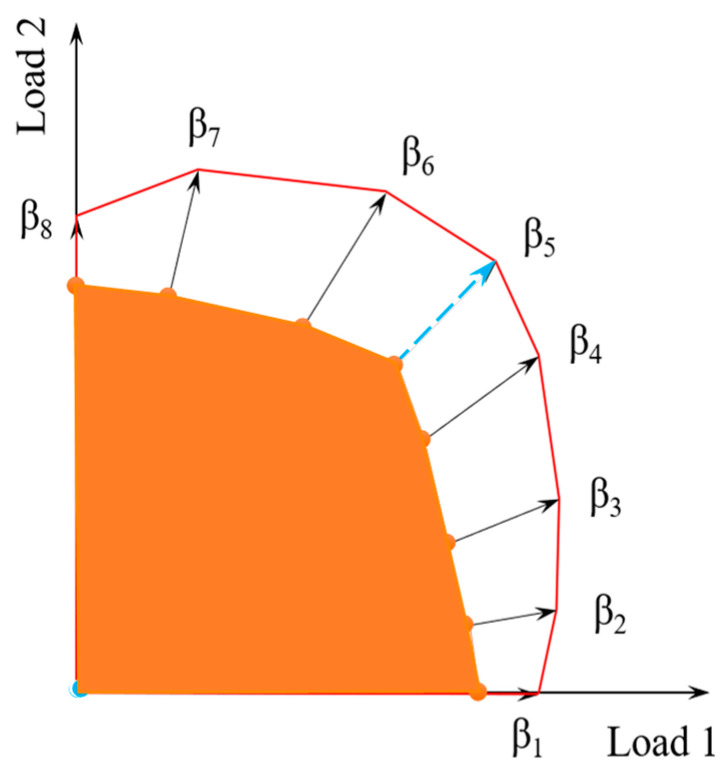
Loading domain and loading path.

Since most of the existing shakedown studies just consider the pressure-bearing structures under constant pressure and cyclic thermal loading, in this study a modified numerical procedure for the shakedown analysis of engineering structures subjected to dual cyclic loadings was developed. In the proposed shakedown analysis procedure, the Abdalla method was further developed by making a distinction between constant loadings and cyclic loadings, while a concept of loading ratio was also introduced. Furthermore, two cyclic loading types were applied along each loading path to calculate the residual stress field. The proposed procedure can be divided into the following five steps:

1. Establish an initial polygonal loading domain, in which the domain corners are corresponding to different loading ratios, as shown in Figure 2.
2. Apply two cyclic loadings on the structure monotonically along each loading path determined by a certain loading ratio, and calculate the elastic stress field of the structure.
3. Increase the maximum loading (*T*_0_ + ∆*T* and *P*_0_ + ∆*P*) incrementally along the loading path in N steps, and calculate the elastic-plastic stress field of the structure at each step.
4. Then assume that the total stress field consists of the elastic stress field and residual stress field. Calculate the residual stress field at each step by removing the elastic stress field, and output the maximum loading value of the residual stress field not exceeding the yield strength. Then the corresponding elastic shakedown limit load under the loading ratio is determined.
5. Change the loading path for different loading ratios and repeat steps 2 to 4 in order to determine the elastic shakedown loading region of the structure under dual cyclic loadings as the orange region shown in Figure 2.

In this study, a square loading domain was established according to the shakedown limit loads under single cyclic internal pressure and single cyclic thermal loading. The maximum combined load is taken as the value of the coordinates on the square boundary. The accuracy of the obtained results can be only affected by the increment of ∆*P* and ∆*T* at each step. In addition, it needs to be noted that the maximum combined load should exceed the yield strength of the structure.

### 3.2. Finite Element Model and the Cyclic Loadings

**Figure 3:**
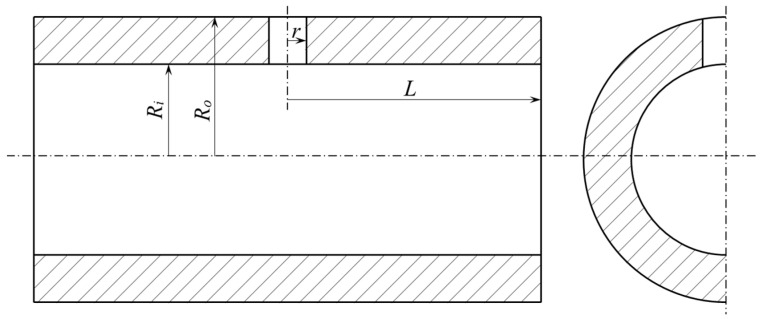
Geometry model.

**Figure 4:**
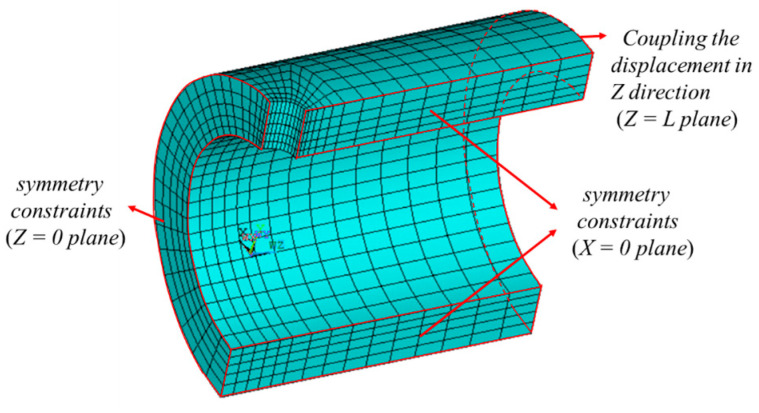
Finite element model.

In this study, 316 stainless steel thick cylindrical vessels with crossholes (TCVCs) were considered for the shakedown analysis. The elastic-perfectly plastic constitutive model was utilized to simulate the shakedown behavior of the TCVC subjected to dual cyclic loadings. The material property parameters of 316 stainless steel at different temperatures are given in Table 2. The initial geometric dimensions of the TCVC are shown in Figure 3, for which the inner radius *R_i_* = 300 mm, outer radius *R_o_* = 450 mm, half length of the whole model *L* = 800 mm, and crosshole radius *r* = 60 mm. Considering the structure geometry and loading symmetry, a quarter of the TCVC is adopted for modeling, as shown in Figure 4. For the quarter finite element model, the symmetry constraints are applied on the planes of *X* = 0 and *Z* = 0, while the coupling displacement is applied on the end plane of the TCVC with *Z* = *L* in the *Z* direction.

**Figure 5:**
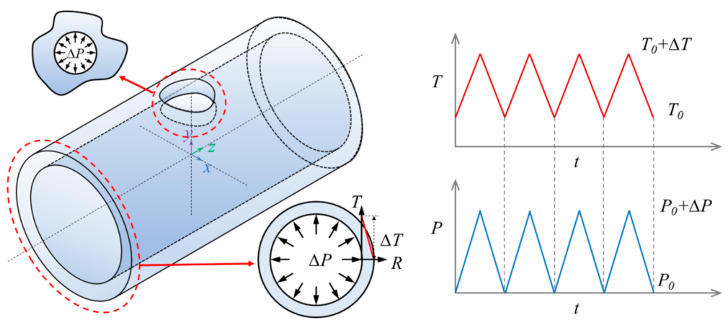
Loading conditions.

The loading conditions of the finite element model primarily include a cyclic internal pressure loading and a cyclic thermal loading, as shown in Figure 5. The cyclic pressure varies from *P*_0_ to *P*_0_ + ∆*P*, and is applied as the inner pressure of the TCVC with an initial internal pressure of *P*_0_ = 0 MPa. When the environmental pressure remains *P*_0_, ∆*P* means the maximum pressure difference between the inner surface and the outer surface of the TCVC. Then the cyclic thermal loading is distributed linearly along the thickness with a constant outer surface temperature of *T*_0_ and a cyclic inner surface temperature of *T*_0_ + ∆*T* (*T*_0_ = 300 °C), in which ∆*T* denotes the maximum temperature difference between the inner surface and the outer surface of the TCVC. Hence, the elastic shakedown limit load can be characterized jointly by the maximum pressure difference ∆*P* and temperature difference ∆T. Furthermore, it should be mentioned that for the condition of TCVC subjected to cyclic thermomechanical loadings, Formula (8) must be used to limit the sum of the primary plus secondary stress to within two times the yield strength in order to ensure the shakedown of the structure.

## 4. Results and Discussion

### 4.1. Method Comparison

**Figure 6:**
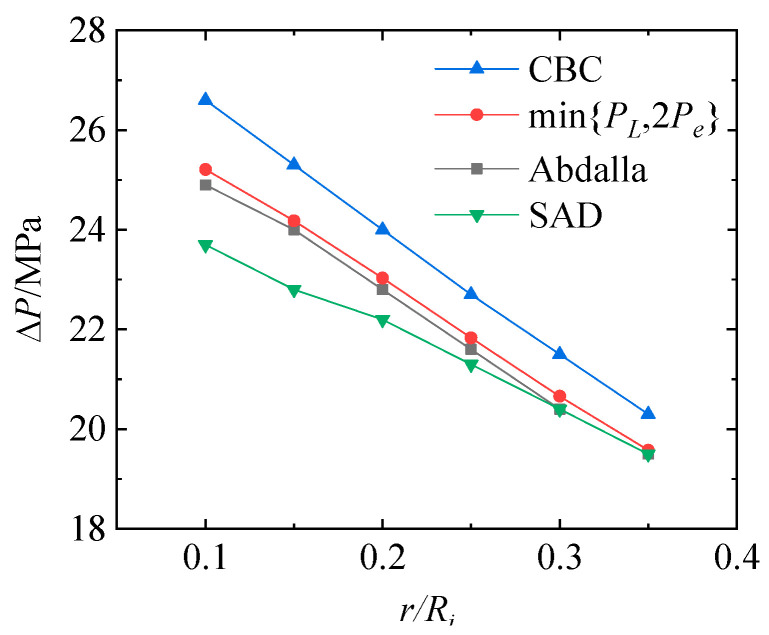
Elastic shakedown limits of the TCVC under cyclic internal pressure.

In order to compare the effectiveness of different shakedown analysis methods, the shakedown analysis of the TCVC subjected to only the cyclic internal pressure was first carried out, as shown in this section. Figure 6 shows the elastic shakedown limits for the structure with different crosshole radii r obtained by the min{*P_L_*, 2*P_e_*} method, Abdalla method, stress analysis design method (SAD) and cycle-by-cycle method (CBC). With the comparison with the results from the cycle-by-cycle method, the most fundamental and accurate method, the most accurate to least accurate are shown to be: min{*P_L_*, 2*P_e_*} method, Abdalla method, and stress analysis design method. In comparison with the stress analysis design method, the results obtained by the min{*P_L_*, 2*P_e_*} method and Abdalla method are more accurate. Meanwhile, compared with the min{*P_L_*, 2*P_e_*} method, the Abdalla method can be used to further analyze the shakedown of structures under non-proportional loadings, which has contributed to its widespread adoption.

### 4.2. The Shakedown Analysis Considering Dual Cyclic Loadings

**Figure 7:**
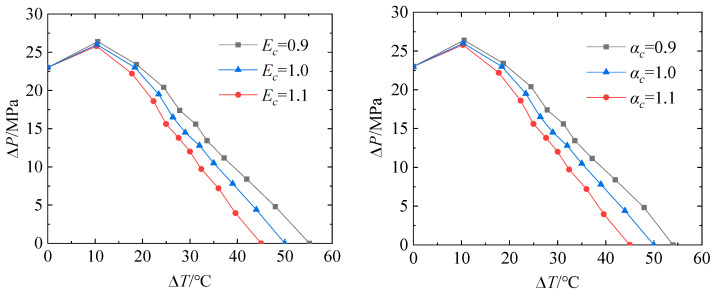
Elastic shakedown limit of TCVC considering different elastic modulus and thermal expansion coefficient.

**Figure 8:**
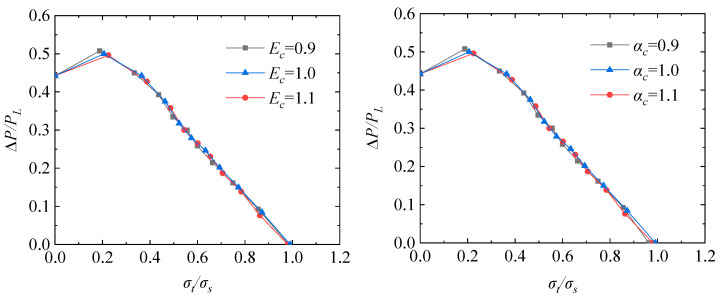
Elastic shakedown limit after dimensionless treatment.

Based on the proposed shakedown analysis flow, the elastic shakedown limit of the TCVC with different material parameters and crosshole radius under dual cyclic loadings are investigated by taking the cyclic thermal loading into account. Firstly, the elastic modulus and thermal expansion coefficient were multiplied by different coefficients *E_c_* and *α_c_* on the original basis to study the effects of material parameters on the elastic shakedown limit of the TCVC. In the simulations, 11 loading ratios were adopted. The simulated results are shown in Figure 7. It can be found that when the yield strength is constant, the elastic shakedown limit load decreases with the increase in elastic modulus and thermal expansion coefficient. In order to conduct an in-depth study on the shakedown of the TCVC, the dimensionless treatment was carried out for Figure 7. In the dimensionless treatment, the maximum pressure difference ∆*P* is divided by the limit pressure *P_L_* of the TCVC as the vertical coordinate, and the elastic thermal loading $\sigma_{t}=E\alpha \Delta T/2(1-v)$ divided by the yield stress *σ_s_* replaces ∆*T* as the horizontal coordinate. Figure 8 shows the results of dimensionless treatment. It can be seen from the figures that the elastic shakedown limits of the TCVC with different material parameters almost coincide, and a general rule of shakedown for the TCVC at a certain size is obtained.

**Figure 9:**
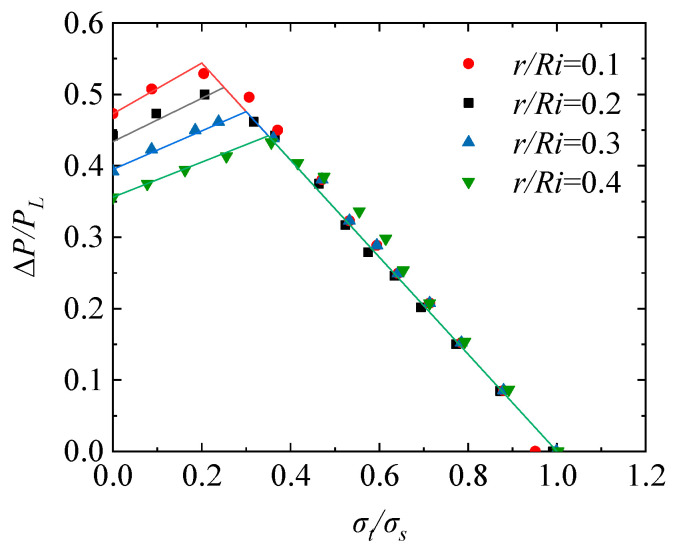
Elastic shakedown limit of the TCVC considering different crosshole radii.

Based on the research above, the effect of different crosshole radii (*r/R_i_* = 0.1, 0.2, 0.3, 0.4) on the elastic shakedown limit of the TCVC with a constant inner radius is analyzed. As an illustration, the model for *r/R_i_* = 0.2 in Figure 4 is used and the crosshole radius changes to 30 mm, 90 mm, and 120 mm. In order to establish the shakedown region in Bree’s diagram, the elastic shakedown limit of the TCVC with 13 loading ratios is calculated based on the proposed numerical flow. The simulated results are shown in Figure 9. The elastic shakedown limit can be approximately expressed by piecewise linear functions. It can be seen from Figure 9 that when *σ_t_/σ_s_* is small, the elastic shakedown limit decreases as the crosshole radius increases; however, when *σ_t_/σ_s_* reaches a specific value, the crosshole radius has little effect on the elastic shakedown limit.

In summary, after the normalization of Bree’s diagram considering the material parameters, the elastic shakedown limit of TCVC in Figure 9 still receives a dispersion of the crosshole radius influence. In order to obtain a more flexible shakedown limit description, we performed a segmented linear fitting to the shakedown boundary of the TCVC for different crosshole radii in Figure 9, and the resulting equation is listed as following:

(10) $$\left\{\begin{array}{l} r/R_{i}=0.1;\Delta P/P_{L}=\left\{\begin{array}{l} 0.355\times \frac{\sigma_{t}}{\sigma_{s}}+0.473\left(\sigma_{t}/\sigma_{s}\leq 0.2\right)\\ -0.68\times \frac{\sigma_{t}}{\sigma_{s}}+0.68\left(0.2<\sigma_{t}/\sigma_{s}\leq 1\right) \end{array}\right.\\ r/R_{i}=0.2;\Delta P/P_{L}=\left\{\begin{array}{l} 0.304\times \frac{\sigma_{t}}{\sigma_{s}}+0.434\left(\sigma_{t}/\sigma_{s}\leq 0.25\right)\\ -0.68\times \frac{\sigma_{t}}{\sigma_{s}}+0.68\left(0.25<\sigma_{t}/\sigma_{s}\leq 1\right) \end{array}\right.\\ r/R_{i}=0.3;\Delta P/P_{L}=\left\{\begin{array}{l} 0.270\times \frac{\sigma_{t}}{\sigma_{s}}+0.395\left(\sigma_{t}/\sigma_{s}\leq 0.3\right)\\ -0.68\times \frac{\sigma_{t}}{\sigma_{s}}+0.68\left(0.3<\sigma_{t}/\sigma_{s}\leq 1\right) \end{array}\right.\\ r/R_{i}=0.4;\Delta P/P_{L}=\left\{\begin{array}{l} 0.245\times \frac{\sigma_{t}}{\sigma_{s}}+0.356\left(\sigma_{t}/\sigma_{s}\leq 0.35\right)\\ -0.68\times \frac{\sigma_{t}}{\sigma_{s}}+0.68\left(0.35<\sigma_{t}/\sigma_{s}\leq 1\right) \end{array}\right. \end{array}\right.$$

Further, it can also be seen from Figure 9 that the shakedown limit value corresponding to *σ_t_/σ_s_* = 0 and the slope of the linear function before the turning point are related to the crosshole radius ratio *r/R_i_*, which can be fitted as a formula as:

(11) $$\begin{array}{r} \Delta P/P_{L}=\frac{0.05\times r/R_{i}+0.066}{0.5\times r/R_{i}+0.15}\times \frac{\sigma_{t}}{\sigma_{s}}+\left(-0.39\times r/R_{i}+0.512\right)\\ \left(\sigma_{t}/\sigma_{s}\leq \left(0.5\times r/R_{i}+0.15\right)\right) \end{array}$$

Therefore, the uniform description of the elastic shakedown limit of the TCVC considering different crosshole radii can be expressed as:

(12) $$\Delta P/P_{L}=\left\{\begin{array}{l} \frac{0.05\times r/R_{i}+0.066}{0.5\times r/R_{i}+0.15}\times \frac{\sigma_{t}}{\sigma_{s}}+\left(-0.39\times r/R_{i}+0.512\right)\\ \;\left(\sigma_{t}/\sigma_{s}\leq \left(0.5\times r/R_{i}+0.15\right)\right)\\ -0.68\times \frac{\sigma_{t}}{\sigma_{s}}+0.68\\ \;\left(\left(0.5\times r/R_{i}+0.15\right)<\sigma_{t}/\sigma_{s}\leq 1\right) \end{array}\right.$$

**Figure 10:**
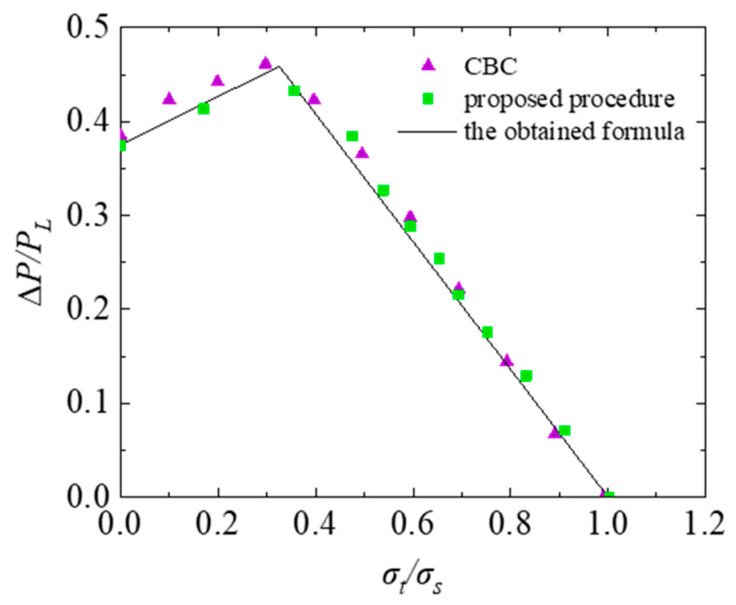
Verification of the obtained formula and the proposed procedure.

In order to validate the reliability of the above fitting formula, the shakedown limit boundary obtained from Formula (12) is compared with the numerical solution result at *r/R_i_* = 0.35 simulated with the finite element method, as shown in Figure 10. It can be seen from Figure 10 that the calculated results of the formulation match well with the finite element analysis results obtained from the proposed procedure. Meanwhile, the shakedown limit boundary determined by the cycle-by-cycle (CBC) method is also shown in Figure 10, which agrees well with that obtained from the proposed procedure. Therefore, the proposed numerical procedure for the shakedown analysis of the TCVC under dual cyclic loadings established in this study is verified to be effective and reliable.

## 5. Conclusions

In this work, a developed numerical procedure for the shakedown analysis of TCVCs under dual cyclic loadings is proposed, and the shakedown behavior of TCVCs under dual cyclic loadings is studied to verify the effectiveness and reliability of the proposed procedure. It is shown that:

(1) Comparing different shakedown analysis methods for only the application of cyclic internal pressure, it is shown that the Abdalla method is more accurate and is also effective for non-proportional loading.
(2) The elastic shakedown limit load of the TCVC decreases with the increase in the elastic modulus and the thermal expansion coefficient of the material. After dimensionless treatment, the influence of material parameters can be eliminated.
(3) The modified shakedown evaluation method and procedure proposed in this work can be used to accurately evaluate the shakedown of TCVCs under cyclic internal pressure and cyclic thermal loading.

## Figures and Tables

**Table 1:** Comparison of different methods.

Method		Supported Load	Main Features
Elastic compensation method		Proportional load	Large error for complex structures
The cycle-by-cycle method		All kinds of load	Time-consuming
Nonlinear superposition methods	Muscat and Mackenzie method	All kinds of load	Only the mechanical loads
	Min {*P_L_*, 2*P_e_*} method	Proportional load	Good precision
	Abdalla method	All kinds of load	Good precision

**Table 2:** Material property parameters of 316 stainless steel.

Temperature	300 °C	400 °C
Young’s modulus (GPa)	176	168
Yield stress (MPa)	109.12	100.80
Poisson’s ratio	0.3	0.3
Thermal expansion coefficient (1/°C)	17.2 × 10^−6^	17.8 × 10^−6^

## Data Availability

Not applicable.
